# Infection, vascularization, remodelling - are stem cells the answers for bone diseases of the jaws?

**DOI:** 10.1186/1746-160X-7-5

**Published:** 2011-02-18

**Authors:** Jörg Handschel, Ulrich Meyer

**Affiliations:** 1 Department for Cranio- and Maxillofacial Surgery, Heinrich-Heine-University, Moorenstr. 5, D-40225 Düsseldorf, Germany

## Abstract

Osteonecrosis after craniofacial radiation (ORN), osteomyelitis and bisphosphonates related necrosis of the jaw (BRONJ) are the predominant bone diseases in Cranio- and Maxillofacial surgery. Although various hypothesis for the pathophysiological mechanisms including infection, altered vascularisation or remodelling exist, the treatment is still a challenge for clinicians. As the classical pharmacological or surgical treatment protocols have only limited success, stem cells might be a promising treatment option, indicated by recently published data.

## 

In maxillofacial surgery clinicians face three diseases of the jaws predominantly: osteonecrosis after craniofacial radiation (ORN), osteomyelitis and bisphosphonates related necrosis of the jaw (BRONJ). Numerous reports exist suggesting various pathological mechanisms and treatment modalities for these diseases [1,2]. Although these publications elucidate the prevalence, risk factors and treatment strategies, they have provided limited data on details of the underlying pathophysiology, especially differences in the three above mentioned diseases. The local or total immunsupressive therapy of many patients (e.g. cancer patients) and the universal presence of hundreds of microorganisms in the oral cavity provide a perfect environment for chronic infections like osteomyelitis. It is unclear if this contributes to BROMJ too. Currently, most evidence exist that the necrotic tissue becomes infected as opposed to the infected tissue becomes necrotic [3]. Regarding the effects on the immune system inconsistent data are reported in the literature. On the one hand bisphosphonates inhibit T lymphocyte activation and proliferation and suppress monocytes production of various pro-inflammatory cytokines [4]. On the other hand they increase the production of pro-inflammatory cytokines by lymphocytes [5]. Whereas the most widely accepted theory to explain the cause of ORN is the theory of hypoxia, radio-induced hypovascularity and hypocellularity [6,7] there is no evidence that the necrotic regions in BRONJ have reduced vasculature or blood supply. However, antiangiogenic effects of bisphosphonates have been reported by other authors [8]. Remoddeling suppression is an other causative factor held responsible for BRONJ despite the fact that there are no published data in humans showing the effects of bisphosphonates on jaw remodeling [2]. Taken together there are only very few studies (e.g. animal studies) clarifying the basic pathophysiological mechanisms of these bone diseases. Very recently, a new treatment modality was introduced elucidating one possible causative factor for BRONJ. Kikuiri and coworkers infused mesenchymal stem cells in BRONJ-like mice. The stem cells modulated the immune system, prevent and cure BRONJ-like disease [9]. Since it is known, that stem cells can induce ectopic bone formation [10] as well as angiogenesis [11], stem cells might be a future treatment option for the above mentioned bone diseases. Particularly, with respect to the full capacity of various stem cell lines [12,13], these cells might become a promising tool for clinicians.
